# Cysteine Supplementation During In Vitro Maturation Enhances Bovine Oocyte Developmental Competence Through Improved Redox Balance and Mitochondrial Function

**DOI:** 10.3390/biology15120973

**Published:** 2026-06-22

**Authors:** Xingyu Zhang, Xin Chen, Ruizhen Jian, Lanting Wang, Size Zhao, Xiaoxuan Fan, Daqing Wang, Guifang Cao

**Affiliations:** 1 College of Veterinary Medicine, Inner Mongolia Agricultural University, Hohhot 010011, China; 2 Animal Embryo and Developmental Engineering Key Laboratory of Higher Education, Institutions of InnerMongolia Autonomous Region, Hohhot 010011, China; 3 Inner Mongolia Autonomous Region Key Laboratory of Basic Veterinary Medicine, Hohhot 010011, China; 4 Inner Mongolia Academy of Agricultural and Animal Husbandry Sciences, Hohhot 010031, China; 5 College of Life Sciences, Inner Mongolia University, Hohhot 010021, China

**Keywords:** oocyte in vitro maturation, L-cysteine, oxidative stress, mitochondrial function, embryo developmental competence

## Abstract

In vitro maturation (IVM) is a critical step affecting the efficiency of bovine in vitro embryo production; however, oxidative stress during in vitro culture can impair oocyte quality and subsequent developmental competence. This study investigated the effects of cysteine supplementation on bovine oocyte IVM, redox homeostasis, mitochondrial status, and transcriptomic changes. Bovine cumulus-oocyte complexes were cultured in IVM medium supplemented with 0, 25, 50, 75, 100, or 125 μM cysteine, and 75 μM was identified as the optimal concentration. Compared with the control group, 75 μM cysteine increased the first polar body extrusion rate from approximately 78% to 81% and improved the fertilization/cleavage rate from approximately 74% to 82%. It also significantly increased the proportions of 2-cell, 4-cell, and 8-cell embryos, whereas morula and blastocyst rates were not significantly affected. At the cellular level, 75 μM cysteine significantly reduced ROS levels and increased GSH content, as indicated by changes in relative fluorescence intensity. JC-1 staining showed that the JC-1 monomer signal decreased from approximately 16.0 to 13.5, whereas the JC-1 aggregate signal increased from approximately 13.2 to 14.8, indicating improved mitochondrial membrane potential status. In addition, lipid droplet fluorescence intensity increased from approximately 11.8 to 13.4, mitochondrial fluorescence intensity increased from approximately 6.0 to 7.0, and cytoskeletal fluorescence intensity showed no significant difference between groups. Smart-seq2 transcriptomic analysis identified 1935 differentially expressed genes, including 1778 upregulated and 157 downregulated genes, which were mainly enriched in translation, ribosomal structural components, RNA binding, oxidative phosphorylation, and metabolism-related pathways. qRT-PCR further confirmed the upregulation of key genes, including NDUFS2, VDAC3, ANXA2, MTHFD1L, and SCD. Overall, 75 μM cysteine improves bovine oocyte IVM quality by enhancing antioxidant capacity, improving mitochondrial membrane potential, increasing lipid-derived energy substrate storage, and regulating genes related to energy metabolism and developmental competence.

## Simple Summary

Supplementation of cysteine during in vitro maturation (IVM) improves bovine oocyte maturation and early embryonic developmental competence by promoting glutathione (GSH) synthesis, scavenging reactive oxygen species (ROS), and restoring mitochondrial function and energy metabolism. Mechanistically, cysteine modulates redox homeostasis and mitochondria-related pathways, thereby alleviating oxidative stress-induced damage and improving cytoplasmic-nuclear synchronization. This strategy provides critical technical support for optimizing livestock in vitro embryo production systems.

## 1. Introduction

In modern animal husbandry, elite breeding serves as the core link to improve production efficiency, optimize product quality and enhance industrial competitiveness. Embryo biotechnology, particularly in vitro embryo production (IVP), enables efficient amplification and precise utilization of superior genetic resources through fine regulation of gametes and early embryos in vitro, and has become one of the vital technical approaches facilitating genetic improvement and large-scale production of livestock such as dairy cattle and beef cattle [1]. The wide application of IVP technology not only accelerates the propagation of elite lines and shortens the generation interval, but also provides an important tool for directional breeding of economically important traits, conservation of rare germplasm resources, disease eradication and population improvement [2].

In the IVP procedure, oocyte in vitro maturation (IVM) is an important step influencing the efficiency of subsequent in vitro fertilization and embryonic development [3]. Compared with the in vivo follicular microenvironment, in vitro culture systems exhibit obvious differences in nutritional composition, endocrine signals, microenvironmental stability and intercellular interactions, which cannot fully mimic the sophisticated intrinsic maturation process of oocytes in vivo [4]. Numerous studies have demonstrated that the developmental competence of in vitro matured oocytes is generally inferior to that of in vivo matured counterparts, accompanied by decreased fertilization rate, cleavage rate, blastocyst formation rate and blastocyst quality [5].

Oocyte quality is the primary determinant of early embryonic developmental progression. As one of the most essential organelles in the oocyte cytoplasm, mitochondria play a decisive role in regulating oocyte maturation quality and developmental competence, acting as the core regulatory hub of cytoplasmic maturation. Mitochondria are the primary site of cellular energy metabolism in oocytes, which generate adenosine triphosphate (ATP) via oxidative phosphorylation to provide sufficient energy for oocyte meiosis initiation, nuclear maturation, cytoplasmic substance synthesis, fertilization and early embryonic cleavage [6]. Meanwhile, mitochondria maintain intracellular redox homeostasis by regulating the generation and elimination of reactive oxygen species (ROS), thereby preventing oocyte oxidative damage induced by excessive ROS accumulation [7]. In addition, mitochondria participate in the regulation of apoptotic signaling pathways. Stable mitochondrial membrane potential and normal function of mitochondrial permeability transition pore can effectively inhibit oocyte apoptosis and ensure normal oocyte maturation and development [8]. During in vivo maturation, oocyte mitochondria undergo dynamic distribution and functional remodeling, migrating from the cortical region to the perinuclear area to form an energy-enriched zone that meets the energy demand of oocyte maturation. By contrast, in in vitro culture, abnormal mitochondrial distribution, decreased membrane potential, insufficient ATP production and impaired ROS scavenging capacity lead to inadequate cytoplasmic maturation, thereby compromising oocyte developmental competence [9].

Oocyte maturation consists of two interrelated and relatively independent processes: nuclear maturation and cytoplasmic maturation [10]. Nuclear maturation refers to the progression of oocyte meiosis from the prophase of the diplotene stage (germinal vesicle, GV stage) to the metaphase of the second meiosis (MII stage) with the extrusion of the first polar body. However, even if oocytes achieve morphological nuclear maturation, insufficient cytoplasmic maturation may result in failure of normal embryonic development after fertilization [11]. Cytoplasmic maturation involves a series of complex physiological changes, including the establishment and maintenance of intracellular redox homeostasis, mitochondrial distribution and functional remodeling, regulation of calcium reserve and calcium signaling, synthesis and storage of maternal factors such as mRNA and protein, reconstruction of lipid and glucose metabolism, as well as organization and dynamic regulation of cytoskeleton [12]. Dysfunction in any of these processes may reduce oocyte developmental competence, manifested as fertilization failure, abnormal cleavage, embryonic diapause and declined embryonic quality.

In in vitro culture, oocytes and surrounding cumulus cells are highly susceptible to oxidative stress. Reactive oxygen species tend to be overproduced and accumulated intracellularly under in vitro conditions, whereas the intrinsic antioxidant system of oocytes possesses limited capacity for ROS elimination [13]. When ROS levels exceed the cellular tolerance threshold, lipid peroxidation, DNA damage and destruction of protein structure and function are triggered, accompanied by decreased mitochondrial membrane potential, restricted ATP generation, cytoskeleton disruption and activation of apoptotic signals, which seriously impair cytoplasmic maturation quality and subsequent embryonic development [14]. Impaired mitochondrial function further exacerbates ROS accumulation, forming a vicious cycle of oxidative stress-mitochondrial damage, which further deteriorates the oocyte maturation microenvironment and reduces developmental competence.

Among various endogenous antioxidants, reduced glutathione (GSH) is one of the most abundant and critical small-molecule antioxidants in oocytes. Glutathione maintains intracellular redox balance by directly scavenging ROS, preserves the reduced state of protein sulfhydryl groups, modulates cellular signaling pathways, protects mitochondrial function, and plays essential roles in sperm decondensation, pronuclear formation and oocyte activation [15]. The capacity of oocytes to synthesize and accumulate adequate GSH during IVM is regarded as a crucial indicator for evaluating cytoplasmic maturation quality and predicting developmental competence. Nevertheless, restricted by medium composition and culture conditions in conventional IVM systems, intracellular GSH levels in in vitro matured oocytes often fail to reach the in vivo level, which further aggravates oxidative damage under in vitro conditions [16].

Against this background, supplementation of antioxidants in IVM medium to improve oocyte cytoplasmic maturation has become an important strategy to enhance IVP efficiency. In the present study, free L-cysteine was selected as the supplement for the IVM culture system mainly because of its direct role in antioxidant metabolism in cumulus–oocyte complexes. L-cysteine is the rate-limiting precursor for GSH synthesis and can be taken up by cumulus cells and oocytes through amino acid transport systems, where it is subsequently used for intracellular GSH biosynthesis. Compared with thiol donors such as N-acetyl-L-cysteine or cysteamine, free L-cysteine can directly participate in GSH synthesis without requiring complex intracellular conversion. Therefore, it is more suitable for evaluating the direct regulatory effect of amino acid supplementation on redox homeostasis and developmental competence in bovine oocytes. Based on this rationale, different concentrations of L-cysteine were added to the bovine oocyte IVM culture system to investigate its effects on oocyte maturation quality and subsequent embryonic developmental competence. Previous studies have indicated that appropriate L-cysteine supplementation in porcine oocyte IVM medium increases intracellular GSH content, eliminates excessive ROS, alleviates oxidative stress-induced cellular damage, optimizes oocyte morphological structure and physiological function, and consequently elevates fertilization rate, cleavage rate and blastocyst formation rate [17]. Recent studies have demonstrated that during in vitro embryo production in cattle, both oocyte maturation and sperm cryopreservation are susceptible to oxidative stress; excessive reactive oxygen species (ROS) can impair sperm quality, oocyte maturation capacity, and in vitro fertilization efficiency [18]. Furthermore, L-cysteine and its metabolites regulate oocyte development through multiple pathways, including modulating mitochondrial energy metabolism to improve ATP supply, affecting lipid metabolism and lipid droplet mobilization to optimize the utilization of energy and membrane structural substances, and regulating epigenetic modification and gene expression profiles to mediate signaling pathways associated with oocyte development.

Although L-cysteine has attracted extensive attention as an antioxidant and GSH precursor in reproductive biology and assisted reproduction, and optimal concentration ranges have been investigated in ovine and porcine oocytes, multiple unresolved issues remain regarding its application in bovine oocyte IVM [19,20]. Given the above concerns, the present study employed bovine oocytes as research objects and established L-cysteine treatment groups with different concentrations based on conventional IVM system to systematically evaluate its effects on oocyte in vitro maturation and subsequent embryonic development. Firstly, from morphological and developmental perspectives, the nuclear maturation rate (polar body extrusion rate), cleavage rate, blastocyst formation rate and blastocyst quality in each group were detected to clarify the effects of different L-cysteine concentrations on oocyte developmental competence. Secondly, at the cellular physiological and biochemical levels, intracellular ROS and GSH levels, mitochondrial membrane potential and distribution, lipid droplet content and distribution pattern, assess the roles of L-cysteine in improving redox balance, maintaining mitochondrial function and regulating lipid metabolism. Finally, high-throughput transcriptomic profiling of oocytes from different groups was performed via Smart-seq2 single-cell RNA sequencing. Differentially expressed genes were screened for functional enrichment and pathway analysis, so as to elucidate the potential molecular mechanism by which L-cysteine regulates oocyte developmental quality at the molecular network level. Meanwhile, during the blastocyst stage, analysis of the expression levels of embryonic totipotency marker genes CDX2, OCT4, and NANOG, along with immunofluorescence staining of apoptosis gene fragments TUNEL, demonstrated the influence of cysteine on blastocyst growth and development.

## 2. Materials and Methods

### 2.1. Materials

Reagents: L-Cysteine (≥98% purity, catalog no. C7352, Sigma-Aldrich, Burlington, MA, USA), in vitro maturation medium (IVM Medium, cat. no. REF 1.03.020, Stroebech Media, Hundested, Denmark), in vitro culture medium (IVC Medium, cat. no. REF 1.07.020, Stroebech Media, Hundested, Denmark), oocyte pickup medium (OPU Medium, cat. no. REF 2.01.500, Stroebech Media, Hundested, Denmark), hyaluronidase (cat. no. H4272, Sigma-Aldrich, Burlington, MA, USA), Medium 199 (cat. no. 11150059, Gibco, Waltham, MA, USA), mineral oil (cat. no. M8410, Sigma-Aldrich, Burlington, MA, USA), Dulbecco’s phosphate-buffered saline (DPBS, cat. no. C3590-0500, Gibco, Waltham, MA, USA), penicillin-streptomycin solution (Double Antibiotics, cat. no. C3420-0100, Gibco, Waltham, MA, USA), reactive oxygen species (ROS) detection kit (cat. no. S0035S, Beyotime Biotechnology, Shanghai, China), CellTracker Blue CMF2HC Dye (CAS no. 215868-45-4, MedChemExpress, Monmouth Junction, NJ, USA), JC-1 staining solution (cat. no. C2003S, Beyotime Biotechnology, Shanghai, China), MitoTracker Red CMXRos (cat. no. C1035, Beyotime Biotechnology, Shanghai, China), Lipid Droplets Staining Kit (cat. no. MAK158, Sigma-Aldrich, Burlington, MA, USA), Cytoskeleton (F-Actin) Staining Kit (cat. no. C1033, Beyotime Biotechnology, Shanghai, China), TUNEL Apoptosis Detection Kit (cat. no. C1088, Beyotime Biotechnology, Shanghai, China), OCT4 immunofluorescence antibody (cat. no. ab181557, Abcam, Cambridge, UK), SOX2 immunofluorescence antibody (cat. no. ab97959, Abcam, Cambridge, UK), NANOG immunofluorescence antibody (cat. no. ab109250, Abcam, Cambridge, UK), goat anti-mouse IgG (H + L) cross-adsorbed secondary antibody, Alexa Fluor™ 488 (cat. no. A32723, Invitrogen, Waltham, MA, USA), and DynaBeads mRNA Direct Kit (cat. no. 610.11, Thermo Fisher Scientific, Waltham, MA, USA) were purchased directly from the manufacturers’ official websites and stored according to the manufacturers’ instructions.

Instruments: Electrophoresis system (Bio-Rad Laboratories, Hercules, CA, USA), pipettes (Eppendorf, Hamburg, Germany), CO_2_ incubator (Thermo Fisher Scientific, USA), benchtop centrifuge (Himac, Hitachi, Tokyo, Japan), refrigerated centrifuge (Thermo Fisher Scientific, USA), fluorescence microscopy imaging system (Olympus Corporation, Tokyo, Japan), and laser scanning confocal microscope (ZEISS LSM 880, Carl Zeiss AG, Oberkochen, Germany).

### 2.2. Oocyte Collection and In Vitro Maturation

Ovaries were obtained from a slaughterhouse in Hohhot. The ovaries were collected and transported back to our laboratory within 2–3 h. Prior to oocyte aspiration, the ovaries were washed three times with physiological saline containing gentamicin until free of blood. Oocytes were then aspirated using sterile, disposable 20 mL syringes with needles to harvest all visible follicles on the surface (diameter 2–8 mm). After aspiration, the contents were gently released into sterile Petri dishes. Cumulus-oocyte complexes (COCs) with intact cytoplasm and surrounded by at least three layers of cumulus cells were selected from the follicular fluid under a stereomicroscope. For in vitro maturation, selected oocytes were washed twice in maturation medium and cultured in 4-well plates (50 oocytes/well) in a CO_2_ incubator (5% CO_2_) at 38.5 °C with maximum humidity for approximately 22 h. A total of 200 oocytes were harvested for each experiment, which was repeated three times with the results averaged.

### 2.3. In Vitro Maturation (IVM) and In Vitro Fertilization (IVF)

IVM: The contents of the syringe were gently released into sterile Petri dishes. Oocytes were selected under a stereomicroscope, washed three times with OPU medium, washed three times with HM199 washing medium, and rinsed once with IVM medium. Oocytes were then cultured in 4-well plates (50 oocytes/well) in a CO_2_ incubator (5% CO_2_) at 38.5 °C with maximum humidity for approximately 22 h. Maturation rates were assessed based on first polar body extrusion relative to the total number of oocytes collected.

IVF: Frozen bovine semen was obtained from Inner Mongolia Saikexing Reproductive Biotechnology (Group) Co., Ltd., Hohhot, China and stored in liquid nitrogen until use. For IVF, frozen semen straws were removed from liquid nitrogen, gently shaken for 3–5 s, and immediately thawed in a 37 °C water bath. After thawing, the straws were wiped dry with sterile absorbent paper. One end of the straw was cut and placed above a sterile centrifuge tube containing 2 mL BO-SemenPrep sperm washing medium. The cotton-plug end was then cut to allow the semen to flow into the BO-SemenPrep medium. Any residual semen remaining in the straw was gently flushed out using a pipette and thoroughly mixed with the washing medium.

Thawed spermatozoa were prepared using a BO-SemenPrep-based centrifugation-washing method. Briefly, the semen suspension was centrifuged at 1500 rpm for 5 min, and the supernatant was removed while leaving a small amount of medium to avoid disturbing the sperm pellet. The sperm pellet was then gently resuspended in 1 mL BO-SemenPrep sperm washing medium and centrifuged again at 1500 rpm for 5 min. After the second wash, the supernatant was discarded, and the final sperm pellet was resuspended in IVF medium. The sperm concentration was adjusted to a final concentration of 1 × 10^6^ sperm/mL before insemination. For IVF, 20 μL of sperm suspension was added to each fertilization droplet and co-incubated with in vitro-matured cumulus-oocyte complexes. Cultivate at 38.5 °C in a three-stage incubator containing 5% O_2_ + 5% CO_2_ + 90% N_2_ for 20 h until fertilization occurs. After fertilization, presumptive zygotes were denuded of cumulus cells by gentle pipetting and transferred into embryo culture medium for further culture.

To determine the dose-dependent effect of cysteine on oocyte in vitro maturation (IVM) and subsequent embryonic development, bovine cumulus-oocyte complexes (COCs) with intact morphology were selected and randomly assigned to six groups: a control group (0 μM cysteine) and five experimental groups supplemented with 25, 50, 75, 100, or 125 μM cysteine. Each group consisted of 200 COCs, and the experiment was replicated three times. Following standard culture for 22 h, the first polar body extrusion rate was assessed to evaluate nuclear maturation. Subsequently, embryos derived from the optimal cysteine concentration group were subjected to in vitro fertilization (IVF) to investigate the impact on early embryonic development.

### 2.4. Effect of Cysteine Concentration on Bovine Oocyte Maturation

To investigate the effect of cysteine on oocyte maturation, different concentrations of cysteine were added to the base IVM medium. Cysteine was directly dissolved in the base IVM medium, filtered through a 0.22 μm membrane for sterilization, aliquoted, and stored at −20 °C. Experimental groups included 0 μM (blank control), 25 μM, 50 μM, 75 μM, 100 μM, and 125 μM cysteine. Cysteine was added at the start of culture and co-incubated with the oocytes. Nuclear maturation was evaluated after culture based on the first polar body extrusion rate.

### 2.5. ROS and GSH Staining

To investigate the effects of 75 μM cysteine on reactive oxygen species (ROS), glutathione (GSH) levels, and mitochondrial membrane potential (MMP) in bovine oocytes, 50 oocytes were randomly selected from both the control and experimental (75 μM cysteine) groups for ROS, GSH, and MMP staining assays. The experiments were replicated three times. Intracellular ROS levels were detected using the fluorescent probe CM-H_2_DCFDA. GSH levels were measured using the CellTracker Blue CMF2HC dye. Mitochondrial membrane potential was assessed using the fluorescent probe JC-1.

Regarding the staining of oocytes, we selected 50 oocytes from each group. For each staining method, we conducted three replicate experiments. For reactive oxygen species (ROS) detection, the ROS Assay Kit was used. Mature denuded oocytes were washed three times with PBS and incubated in working solution containing 5 μM CM-H_2_DCFDA probe at 37 °C in the dark for 30 min. After washing three times with PBS, fluorescence intensity was observed under a laser scanning confocal microscope with excitation at 495 nm and emission at 530 nm to analyze intracellular ROS levels.

For glutathione (GSH) detection, CellTracker Blue CMF2HC Dye was used. Mature denuded oocytes were washed three times with PBS and incubated in 5 μM dye solution at 37 °C for 30 min. After washing three times with PBS, fluorescence intensity was analyzed under a confocal microscope with excitation at 450 nm and emission at 460 nm.

### 2.6. Lipid Drop Staining

Using BODIPY 493/503 (4,4-difluoro-1,3,5,7,8-pentamethyl-4-borano-3A,4A-diazacy-S-indene), mature exposed oocytes were first washed twice in PBS, then transferred to 1 mL of BODIPY 493/503 working solution and incubated under light protection for 20 min at 37 °C. The oocytes were subsequently washed three times with PBS, and the fluorescence intensity of each oocyte was analyzed using a laser confocal microscope with an excitation wavelength of 495 nm, an emission wavelength of 503 nm, and green fluorescence detection.

### 2.7. Cytoskeleton

The mature exposed oocytes were first washed twice with PBS using phalloidin, fixed in 4% polyformaldehyde for 20 min, and then blocked and permeabilized with a 0.5% X-100 + 1% BSA solution. They were washed three times with PBS (totaling five minutes), incubated in phalloidin staining solution at 37 °C for 30 min, followed by three washes with PBS. Subsequently, the oocytes were incubated in DAPI solution at 37 °C for 15 min and washed three times with PBS (totaling five minutes). Under a laser confocal microscope, fluorescence was analyzed using an excitation wavelength of 496 nm and an emission wavelength of 516 nm; green fluorescence was observed, while nuclei exhibited blue fluorescence with a maximum excitation wavelength of 364 nm and a maximum emission wavelength of 454 nm. The fluorescence intensity of each oocyte was quantified.

### 2.8. Mitochondrial Membrane Potential (MMP) and Mitochondrial Staining

For MMP detection, the JC-1 Assay Kit was used. Mature denuded oocytes were washed three times with PBS, pre-warmed JC-1 staining solution was applied, and samples were incubated at 37 °C in the dark for 20 min. After washing three times with PBS, fluorescence was observed under a confocal microscope. JC-1 monomer: excitation 514 nm, emission 529 nm; JC-1 aggregates: excitation 585 nm, emission 590 nm.

For mitochondrial staining, MitoTracker Red CMXRos was used. Mature denuded oocytes were washed three times with PBS, incubated in working solution at 37 °C for 30 min, washed three times with PBS, and observed under a confocal microscope with excitation at 579 nm and emission at 599 nm.

### 2.9. Blastocyst Apoptosis (TUNEL Assay)

The TUNEL Apoptosis Detection Kit was used. Select 30 mature blastocysts, wash them three times with PBS, fix them in 4% polyformaldehyde for 20 min, and then perform permeabilization using 0.5% Triton X-100 and 1% bovine serum albumin (BSA). After washing three times with PBS (5 min total), samples were incubated in biotin-labeled solution at 37 °C for 60 min, washed three times with PBS, and stained with DAPI solution at 37 °C for 15 min. After washing three times with PBS (5 min total), samples were mounted with antifade reagent. Fluorescence was observed under a confocal microscope with excitation at 579 nm and emission at 599 nm for apoptosis (red) and excitation at 364 nm and emission at 454 nm for nuclei (blue).

### 2.10. Immunofluorescence Staining for CDX2, OCT4, and NANOG

Regarding the staining of blastocysts, we selected 15 blastocysts for each group. For each type of staining, we conducted three replicate experiments. Mature blastocysts were washed three times with PBS, fixed in 4% paraformaldehyde for 20 min, and permeabilized with PBS containing 0.5% Triton X-100 and 1% BSA. After washing three times with PBS (5 min total), blastocysts were incubated with primary antibodies at 4 °C overnight. The following day, samples were washed three times with PBS and incubated with corresponding secondary antibodies at 37 °C for 60 min. Nuclei were counterstained with DAPI solution at 37 °C for 15 min. Following a final wash with PBS, samples were mounted with antifade reagent and observed under a laser scanning confocal microscope.

Specific antibodies and fluorescence parameters:

CDX2: CDX2 Monoclonal Antibody; Goat Anti-Mouse IgG (H + L) Cross-Adsorbed Secondary Antibody, Alexa Fluor™ 594 . Excitation/Emission: 590/617 nm (red).

OCT4: OCT4 Recombinant Rabbit Monoclonal Antibody ; Donkey Anti-Rabbit IgG (H + L) Highly Cross-Adsorbed Secondary Antibody, Alexa Fluor™ 488 . Excitation/Emission: 495/519 nm (green).

NANOG: NANOG (D73G4) XP^®^ Rabbit mAb; Goat Anti-Rabbit IgG (H + L) Cross-Adsorbed Secondary Antibody, Alexa Fluor™ 594. Excitation/Emission: 590/617 nm (red).

Nuclear Counterstain: DAPI. Excitation/Emission: 364/454 nm (blue).

Microscopic observation of embryonic development at different time points after in vitro fertilization: By day 2 post-fertilization, the embryos had developed to the two-cell stage; by day 3, they reached the four-cell stage; by day 4, they were at the eight-cell stage; by day 5, a morula was formed; and by day 6, they had developed into blastocysts. On day 2, the number of cleavage embryos was counted, with the cleavage rate calculated as (number of cleavage embryos/total number of IVF embryos) × 100%. The numbers of morules and blastocysts were recorded on days 5 and 6 respectively, and corresponding developmental rates were calculated. Embryos were graded using an inverted microscope based on cleavage sphere uniformity, cytoplasmic integrity, and proportion of cellular debris. Daily records of embryonic developmental progression were maintained, and data from all experimental groups were compiled for statistical analysis.

### 2.11. Smart-Seq2 Single-Cell Sequencing Analysis

Following maturation, oocytes from the control group and the 75 μM cysteine treatment group were selected for comparative analysis. Samples were washed three times in HM199 solution with gentle pipetting to remove adherent granulosa cells, followed by three washes in Ca^2+^/Mg^2+^-free DPBS. All washing steps were performed on a 38.5 °C heating plate within 10 min to maintain viability. Oocytes were transferred into 0.2 mL RNase-free tubes containing 2.5 μL lysis buffer, ensuring carryover wash volume did not exceed 1 μL. Tubes were immediately placed on ice, sealed with parafilm, labeled, and stored at −80 °C. Three independent biological replicates were performed. Samples were shipped on dry ice to Hangzhou Lianchuan Biotechnology Co., Ltd., Hangzhou, China for Smart-seq2 sequencing.

The Smart-seq2 library in this study was constructed using poly(A) enrichment and sequenced on the NovaSeq™ 6000 platform (Illumina, San Diego, CA, USA). Sequencing was performed as paired-end reads with a read length configuration of PE150. Raw sequencing data were quality-controlled using FastQC v0.10.1, and RNA-seq data quality was further assessed with RSeQC v4.0.0. High-quality clean reads meeting downstream analysis requirements were obtained for each sample, with an average sequencing output of approximately 6.45 Gb of clean data, corresponding to about 21.5 million paired-end clean reads. Quality statistics indicated that the proportion of clean reads was approximately 97.2%, Q20 ≥ 97.8%, Q30 ≥ 93.5%, and GC content was around 48.6%. Clean reads were subsequently aligned to the Bos taurus reference genome Bos_taurus. ARS-UCD1.2 using HISAT2 v2.2.1, achieving an average alignment rate of approximately 94.6%. Transcript assembly and merging were performed using StringTie v2.1.6, and differential expression analysis was conducted with DESeq2 v1.22.2. The GO, KEGG, and DO databases were updated in May 2021, and Reactome Version 76 was used.

### 2.12. Gene Expression Analysis in Bovine Oocytes

*NDUFS2* is a key gene involved in the mitochondrial respiratory chain and developmental potential of oocytes [21]. *VDAC3* encodes an outer mitochondrial membrane channel protein and participates in cellular energy metabolism and signal transduction [22]. *ANXA2* is responsible for regulating membrane trafficking and cytoskeleton organization [23]. *MTHFD1L* mediates the production of NADPH [24]. *SCD* participates in fatty acid metabolism and modulates lipid droplet formation and membrane lipid composition in oocytes [25].

Perform reverse transcription on the *NDUFS2*, *VDAC3*, *ANXA2*, *MTHFD1L*, and *SCD* genes. The primer sequences are shown in Table 1. RNA was extracted from mature oocytes using the DynaBeads mRNA Direct Kit. Reverse transcription was performed using the same kit. Briefly, 5 μL of RNA annealing buffer OT was added to 10 μL of extracted RNA and heated at 65 °C for 3 min. After cooling, 9 μL of cDNA synthesis buffer and 1 μL of RNA Script RT/RNase inhibitor mix were added. Reverse transcription was performed at 37 °C for 50 min, followed by 85 °C for 5 min. cDNA was stored at −80 °C. qRT-PCR reactions (20 μL total) contained 1 μL template, 1 μL each of forward and reverse primers, 10 μL enzyme mix, and 7 μL ddH_2_O. Cycling conditions: 95 °C for 3 min; 40 cycles of 95 °C for 15 s, 60 °C for 30 s, 72 °C for 2 s; final extension at 72 °C for 5 min. Relative expression was calculated using the 2^(−ΔΔCt) method.

To clarify the mechanism underlying the regulatory effect of cysteine on oocyte development, mature oocytes from the 75 μM cysteine treatment group and the blank control group (0 μM) were used as experimental materials in this study. The Smart-seq2 single-cell RNA sequencing technology was applied for transcriptomic analysis. The thresholds of |log_2_FC| ≥ 1 and *q* < 0.05 were set as the screening criteria for differentially expressed genes (DEGs) to guarantee the statistical significance and biological implication of the results.

All experiments were repeated at least three times, and data are presented as the mean ± SEM. Statistical differences among multiple groups were analyzed using one-way analysis of variance (ANOVA). When significant differences were detected, multiple comparisons were performed using Tukey’s honest significant difference (Tukey’s HSD) post hoc test. A value of *p* < 0.05 was considered statistically significant.

## 3. Analysis

Fluorescence intensities related to gene expression were quantified using ImageJ 1.8.0 software (National Institutes of Health, Bethesda, MD, USA). Each experiment was independently repeated at least three times. Data are presented as mean ± standard deviation (SD). Statistical analyses were performed using Prism 7 (GraphPad Software, San Diego, CA, USA). Significance was determined by one-way or two-way ANOVA followed by Tukey’s multiple comparison test, as appropriate. *p* < 0.05 was considered statistically significant. Significance levels: ns (not significant, *p* > 0.05), * *p* < 0.05,** *p* < 0.01,*** *p* < 0.001.

Each experimental group in this study included three biological replicates, with technical replicates synchronized within each biological replicate to ensure sample detection stability and data quality control. For downstream statistical analysis, the mean values of biological replicates were used as independent statistical units, while technical replicates were primarily employed to enhance the reliability and reproducibility of the results. Prior to performing one-way ANOVA, normality tests were conducted on the raw data for each measured parameter using the Shapiro–Wilk test to assess whether the data followed a normal distribution, with a significance level set at α = 0.05; data were considered to meet the normality assumption when *p* > 0.05. Subsequently, Levene’s test was applied to further evaluate homogeneity of variances across groups. For data satisfying both normality and homogeneity of variance, standard one-way ANOVA was used to analyze inter-group differences. If data were normally distributed but showed unequal variances, Welch’s ANOVA was applied for adjusted analysis. For non-normally distributed data, appropriate non-parametric tests were used to ensure the scientific validity and reliability of the statistical results.

## 4. Results

### 4.1. Effect of Cysteine on In Vitro Maturation and Embryonic Developmental Potential of Bovine Oocytes

**Figure 1:**
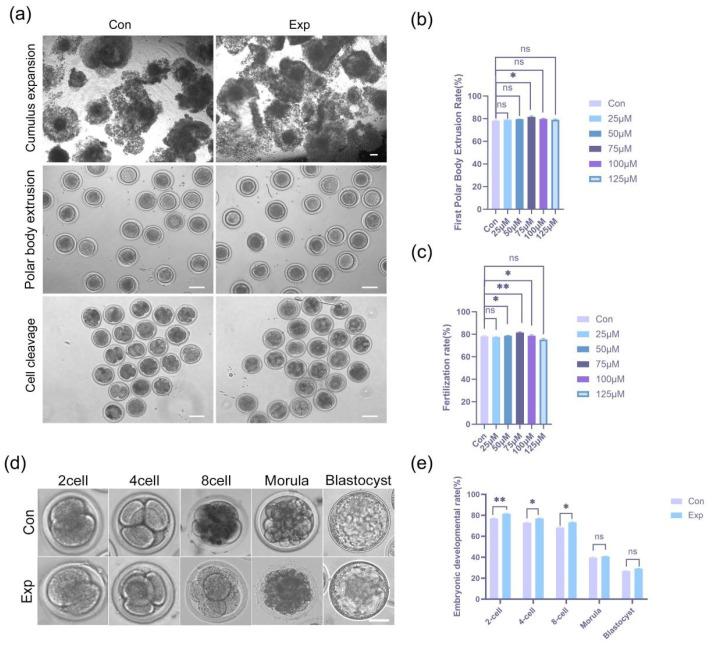
Cysteine supplementation improves bovine oocyte maturation and early embryonic developmental competence. (**a**) Representative morphological images showing cumulus expansion, first polar body extrusion, and early cleavage in oocytes from the control and cysteine-treated groups, Scale bar: 80 μm. (**b**) Quantification of first polar body extrusion rates under different cysteine concentrations, showing that 75 μM cysteine produced the most favorable effect on nuclear maturation. (**c**) Fertilization/cleavage rates after IVF, indicating improved early developmental activation following 75 μM cysteine treatment. (**d**) Representative images of embryos at the 2-cell, 4-cell, 8-cell, morula, and blastocyst stages, Scale bar: 50 μm. (**e**) Quantitative analysis of embryonic developmental rates, showing that cysteine treatment promoted early cleavage-stage development without significantly altering morula or blastocyst rates. Scale bars: 100 μm in (**a**); 50 μm in (**d**). ns, not significant; * *p* < 0.05, ** *p* < 0.01.

The results demonstrated that all experimental groups supported oocyte maturation. Notably, the 75 μM cysteine group exhibited well-dispersed cumulus cells and a significantly higher first polar body extrusion rate compared to the control group (*p* < 0.05, Figure 1b). Concentrations exceeding 75 μM did not confer additional benefits. In the IVF cleavage assay, the 75 μM group showed a markedly higher cleavage rate than the control group (*p* < 0.01, Figure 1c) and significantly improved developmental rates to the 4-cell and 8-cell stages (*p* < 0.05, Figure 1e). Furthermore, embryos in the 75 μM group developed normally to the blastocyst stage without morphological abnormalities, although the overall blastocyst rate did not differ significantly from that of the control group.

### 4.2. Effect of 75 μM Cysteine on ROS, GSH, and Mitochondrial Membrane Potential (MMP) in Bovine Oocytes

**Figure 2:**
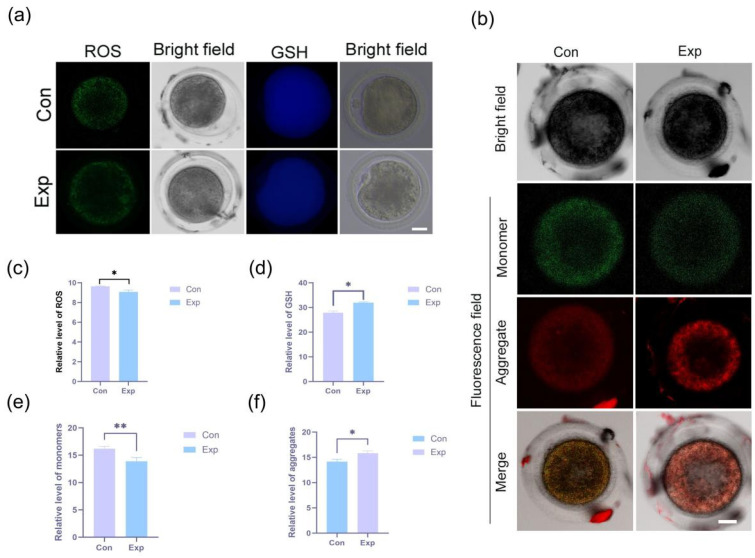
Cysteine enhances redox homeostasis and improves mitochondrial membrane potential during bovine oocyte maturation. (**a**) Representative fluorescence images of ROS and GSH in oocytes from the control and 75 μM cysteine-treated groups. Reduced ROS fluorescence and enhanced GSH fluorescence indicate that cysteine alleviated oxidative stress and strengthened antioxidant capacity, Scale bar: 25 μm. (**b**) Representative JC-1 staining images showing mitochondrial membrane potential status. Increased JC-1 aggregate fluorescence and reduced monomer fluorescence suggest improved mitochondrial membrane potential in cysteine-treated oocytes, Scale bar: 25 μm. (**c**–**f**) Quantification of ROS, GSH, JC-1 monomer, and JC-1 aggregate fluorescence intensities. These results indicate that 75 μM cysteine maintains redox balance and mitochondrial membrane potential during IVM. Scale bars: 25 μm. * *p* < 0.05, ** *p* < 0.01.

The results indicated that cysteine significantly improved the redox homeostasis of oocytes. Compared with the control group, the relative ROS level in the 75 μM cysteine group was significantly decreased (*p* < 0.05, Figure 2c), while the GSH content was significantly increased (*p* < 0.05, Figure 2d). This confirms that cysteine inhibits excessive ROS accumulation and elevates the antioxidant GSH content.

**Figure 3:**
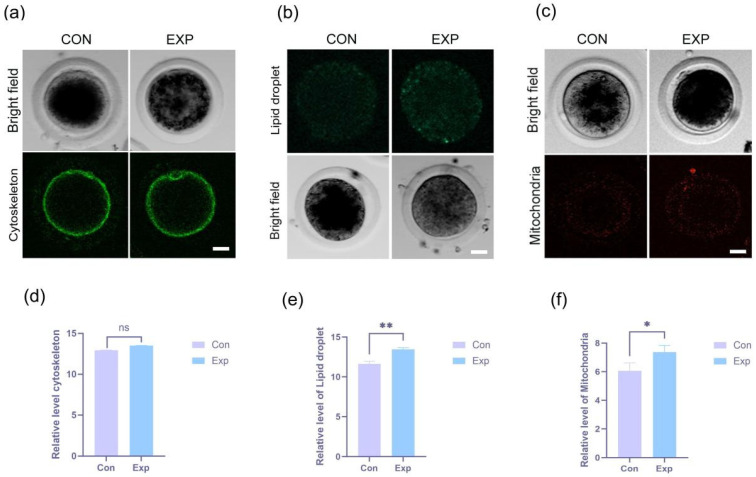
Cysteine increases lipid-derived energy storage and mitochondrial-associated homeostasis without disrupting cytoskeletal organization. (**a**) Representative fluorescence images of cytoskeletal organization in control and 75 μM cysteine-treated oocytes, Scale bar: 25 μm. (**b**) Representative images of lipid droplets, showing increased lipid droplet accumulation after cysteine treatment, Scale bar: 25 μm. (**c**) Representative images of mitochondrial staining, showing enhanced mitochondrial fluorescence in the cysteine-treated group, Scale bar: 25 μm. (**d**–**f**) Quantitative analysis of cytoskeleton, lipid droplet, and mitochondrial fluorescence intensities. The results indicate that 75 μM cysteine increased lipid-derived energy substrate storage and mitochondrial-associated signals while maintaining cytoskeletal integrity. Scale bars: 25 μm. ns, not significant; * *p* < 0.05, ** *p* < 0.01.

Mitochondrial function assessment further revealed that 75 μM cysteine significantly improved oocyte MMP. Compared with the control group, the relative level of MMP aggregates (J-aggregates) in the experimental group was significantly increased (*p* < 0.05), while the relative level of the depolarized monomeric form (JC-1 monomer) was markedly decreased (*p* < 0.01, Figure 3e,f). These findings suggest that cysteine maintains mitochondrial membrane potential status by reducing the degree of mitochondrial depolarization.

### 4.3. Effect of 75 μM Cysteine on the Cytoskeleton, Lipid Droplets, and Mitochondria of Bovine Oocytes

Regarding the cytoskeleton, no significant difference in relative fluorescence intensity was observed between the 75 μM cysteine group and the control group (*p* > 0.05, Figure 3a), indicating that cysteine does not adversely affect cytoskeletal organization. In contrast, the lipid droplet content in the 75 μM cysteine group was markedly elevated compared to the control (*p* < 0.01, Figure 3e).

Mitochondrial analysis revealed that the relative mitochondrial content in the 75 μM cysteine group was significantly higher than that in the control group (*p* < 0.05, Figure 3f). These results further confirm that supplementation with 75 μM cysteine enhances mitochondrial content and may be associated with altered metabolic activity in bovine oocytes.

### 4.4. Effect of 75 μM Cysteine on Blastocyst Marker Gene Expression and Apoptosis

**Figure 4:**
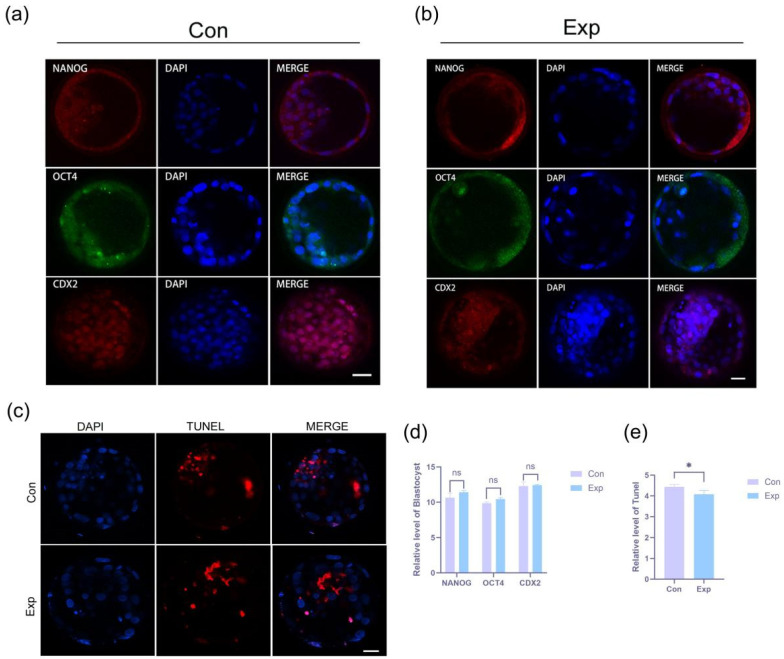
Cysteine maintains blastocyst lineage marker expression and reduces apoptosis. (**a**,**b**) Representative immunofluorescence images of NANOG, OCT4, and CDX2 in blastocysts derived from control and 75 μM cysteine-treated oocytes. Comparable expression patterns indicate that cysteine treatment did not disrupt pluripotency- or trophectoderm-related lineage marker expression, Scale bar: 25 μm. (**c**) Representative TUNEL staining images of blastocysts from both groups, Scale bar: 25 μm. (**d**) Quantitative analysis of NANOG, OCT4, and CDX2 fluorescence intensities. (**e**) Quantification of TUNEL-positive signals, showing that cysteine treatment reduced blastocyst apoptosis. These results suggest that cysteine supports embryo quality without impairing lineage specification. Scale bars: 25 μm. ns, not significant; * *p* < 0.05.

The effect of cysteine on blastocyst formation was evaluated by detecting embryonic totipotency markers. Compared with the control group, the relative fluorescence intensities of NANOG, OCT4, and CDX2 in blastocysts derived from the 75 μM cysteine group showed no significant differences (*p* > 0.05, Figure 4d). These results indicate that cysteine does not alter the overall expression levels of core pluripotency (NANOG/OCT4) and trophectoderm (CDX2) genes, nor does it disrupt embryonic cell lineage differentiation capacity.

Furthermore, TUNEL assay results demonstrated that supplementation with 75 μM cysteine significantly reduced blastocyst apoptosis. Compared with the control group, the relative level of TUNEL-positive cells in the experimental group was markedly decreased (*p* < 0.05, Figure 4e). This suggests that cysteine enhances the anti-apoptotic capacity of embryos and improves overall embryo quality by reducing apoptosis at the blastocyst stage.

### 4.5. Smart-Seq2 Sequencing Analysis of Bovine MII Oocytes Treated with Cysteine

**Figure 5:**
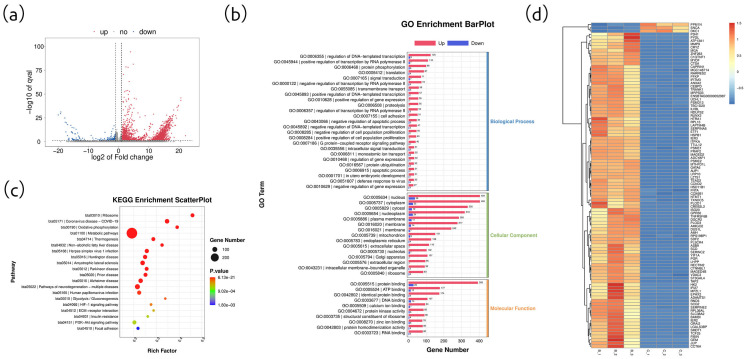
Transcriptomic profiling reveals cysteine-induced activation of genes associated with protein synthesis, mitochondrial metabolism, and redox-related regulation. (**a**) Volcano plot showing differentially expressed genes between control and 75 μM cysteine-treated MII oocytes. (**b**) GO enrichment analysis showing that differentially expressed genes were mainly associated with translation, RNA binding, ribosomal structural components, cytoplasm, and membrane-related components. (**c**) KEGG enrichment analysis indicating enrichment of ribosome, oxidative phosphorylation, and metabolism-related pathways. (**d**) Hierarchical clustering heatmap showing distinct transcriptomic profiles between the control and cysteine-treated groups. These results suggest that cysteine supplementation promotes a transcriptional state associated with restored redox balance, protein synthesis, and metabolic activity.

The sequencing results revealed that a total of 1778 DEGs were significantly upregulated and 157 DEGs were markedly downregulated in the 75 μM cysteine group compared with the 0 μM control group (Figure 5a). Smart-seq2 transcriptomic analysis showed that the differentially expressed genes in the experimental group were predominantly upregulated, suggesting that amino acid supplementation did not induce broad transcriptional repression but may promote the recovery of transcriptional activity in oocytes. GO enrichment analysis indicated that these genes were mainly associated with translation, RNA binding, ribosomal structural components, cytoplasm, and membrane-related components. KEGG analysis further showed enrichment in ribosome, oxidative phosphorylation, and metabolism-related pathways. These results suggest that the asymmetry between upregulated and downregulated genes may be related to reduced oxidative stress and restored redox homeostasis, reflecting a transition of oocytes from a stress-responsive state toward a relatively stable and metabolically active state(Figure 5b). The core differential genes included NDUFS2, VDAC3, ANXA2, MTHFD1L and SCD, suggesting that cysteine supplementation may modulate the above pathways and gene expression to regulate the developmental progression of oocytes.

### 4.6. Effect of Cysteine on the Expression of Genes Related to Oocyte Growth and Apoptosis

**Figure 6:**
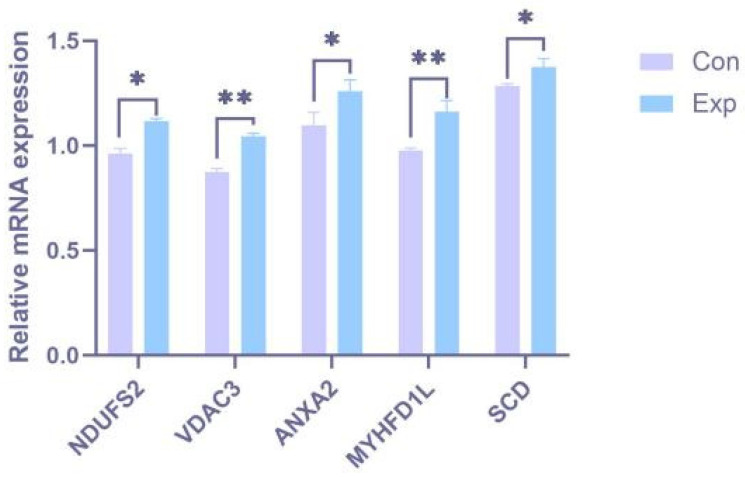
qRT-PCR validation confirms the upregulation of key genes related to mitochondrial metabolism, redox regulation, cytoskeletal organization, and lipid homeostasis. Relative mRNA expression levels of NDUFS2, VDAC3, ANXA2, MTHFD1L, and SCD in control and 75 μM cysteine-treated oocytes. The upregulation of these genes supports the transcriptomic findings and suggests that cysteine may regulate oocyte maturation through mitochondrial energy metabolism, metabolite exchange, cytoskeletal-associated processes, NADPH production, and lipid-derived energy storage. * *p* < 0.05, ** *p* < 0.01.

To verify the expression patterns of differentially expressed genes screened by Smart-seq2 sequencing, the relative mRNA levels of NDUFS2, VDAC3, ANXA2, MTHFD1L and SCD were determined in this study. The results showed that the mRNA expression levels of all these genes were significantly upregulated in the experimental group relative to the control group. Among them, NDUFS2, ANXA2 and SCD were significantly differentially expressed (*p* < 0.05), while VDAC3 and MTHFD1L presented an extremely significant difference (*p* < 0.01, Figure 6. VDAC3 upregulation may enhance mitochondrial energy and metabolite exchange, thereby providing metabolic support for meiotic progression. Together with the cytoskeletal fluorescence staining results in this study, VDAC3 upregulation may be associated with the maintenance of cytoskeletal stability and improved chromosome alignment. However, because VDAC3 protein expression and functional intervention were not examined, its specific role requires further validation. This study further confirmed that cysteine affects oocyte development-related pathways by regulating the expression of the above genes.

## 5. Discussion

This study aimed to explore the effects of exogenous cysteine on the in vitro maturation (IVM) quality and subsequent developmental competence of bovine oocytes. Through systematic concentration screening and multiple functional analyses, this study confirmed that supplementation with 75 μM cysteine in IVM medium effectively improved the maturation rate and cytoplasmic maturation quality of bovine oocytes. By regulating redox homeostasis, mitochondrial function and the expression of key genes, cysteine ultimately promoted the formation of embryos with superior developmental potential.

Oxidative stress is a core limiting factor for the in vitro maturation quality of oocytes. After leaving the physiological antioxidant microenvironment of ovarian follicles, oocytes are prone to excessive accumulation of reactive oxygen species (ROS) under atmospheric oxygen conditions in vitro. Excess ROS further induces lipid peroxidation, DNA damage and mitochondrial dysfunction, ultimately leading to defective cytoplasmic maturation and reduced fertilization capacity as well as embryonic developmental potential [26]. As the rate-limiting precursor for intracellular glutathione (GSH) biosynthesis, the antioxidant property of cysteine has been validated in oocyte studies of multiple species. As early as 1995, de Matos et al. confirmed that supplementation of sulfhydryl compounds in bovine IVM medium significantly increased intracellular GSH content and further improved embryonic developmental rates [27]. Subsequent studies in pigs [18], sheep [19] and mice [28] further verified that exogenous cysteine enhances endogenous GSH synthesis, eliminates excessive ROS, and alleviates oxidative stress damage in oocytes. The present results are highly consistent with the above classical findings. Treatment with 75 μM cysteine markedly reduced intracellular ROS levels and increased GSH accumulation in bovine oocytes, confirming that cysteine reconstructs the GSH-dependent antioxidant defense system and maintains a stable redox microenvironment for bovine oocyte IVM. Through a series of concentration gradient pre-experiments, 75 μM was identified as the optimal supplemental concentration for bovine oocyte IVM. This concentration avoided insufficient antioxidant effects at low doses and potential cellular microenvironment disturbance induced by high-dose sulfhydryl compounds, providing an accurate and repeatable dosage reference for the standardized application of cysteine in bovine IVM systems.

As the cellular powerhouse and core regulator of metabolism, mitochondrial biogenesis, membrane potential homeostasis and oxidative phosphorylation efficiency are critical determinants of cytoplasmic maturation, meiotic progression and subsequent embryonic developmental potential of oocytes [29]. Previous studies in porcine oocyte IVM models demonstrated that exogenous cysteine alleviates oxidative stress injury and maintains mitochondrial membrane potential (MMP) homeostasis [30]. In mouse aged oocyte models, the cysteine derivative N-acetyl-L-cysteine (NAC) effectively improved mitochondrial respiratory function and restored oocyte developmental competence [31]. JC-1 staining was used to evaluate mitochondrial membrane potential. Compared with the control group, the treatment group showed a higher red/green fluorescence ratio, suggesting that cysteine supplementation helped maintain mitochondrial membrane potential and reduce mitochondrial depolarization. In addition, MitoTracker staining indicated a more stable mitochondrial staining pattern in the treatment group, suggesting that cysteine supplementation may contribute to the maintenance of mitochondrial distribution and mitochondrial membrane-associated homeostasis. Compared with previous studies, the innovation of this study lies in providing transcriptomic evidence of how cysteine regulates mitochondrial function in bovine oocytes at the transcriptomic level. Transcriptomic sequencing revealed that differentially expressed genes in cysteine-treated oocytes were significantly enriched in energy metabolism-related pathways, including oxidative phosphorylation and ATP binding. The expression of NDUFS2, encoding a subunit of mitochondrial respiratory chain complex I, and VDAC3, encoding an outer mitochondrial membrane channel protein, were both significantly upregulated. These results indicate that cysteine may coordinately improve mitochondrial biogenesis and functional activity by upregulating energy metabolism-related genes, thereby providing adequate energy supply for oocyte maturation, fertilization and early embryonic development.

Lipid droplets are important lipid storage structures in oocytes and represent a major form of intracellular energy reserve. In the present study, an increased number of lipid droplets, together with elevated stearoyl-CoA desaturase (SCD) activity, was observed in the treated oocytes, suggesting enhanced storage capacity of lipid-derived energy substrates. Increased SCD activity may contribute to the regulation of fatty acid desaturation and lipid homeostasis, thereby helping to maintain the energy reserve status during oocyte maturation. Therefore, the increased lipid droplet accumulation observed in this study mainly reflects enhanced energy substrate storage in oocytes, rather than increased lipolysis or fatty acid β-oxidation. Moreover, the expression of ANXA2, which participates in membrane trafficking and cytoskeleton regulation, was also altered. Although no significant difference in cytoskeleton fluorescence intensity was observed, the upregulation of ANXA2 validated by Smart-seq2 and RT-qPCR indicated that cysteine exerts a potential regulatory role in maintaining cellular structure stability and material transport in oocytes. However, transcript-level changes do not necessarily indicate corresponding alterations in protein expression or mitochondrial function. Since key mitochondrial proteins such as NDUFS2 were not validated by Western blot or immunofluorescence in this study, the current results only indicate changes in mitochondrial-related transcriptional activity and cannot directly demonstrate corresponding changes at the protein or functional level.

Cysteine improves blastocyst quality and developmental competence. Experimental results showed that after in vitro fertilization of 75 μM cysteine-matured oocytes, the TUNEL-based apoptotic level of blastocysts was significantly decreased. This may be attributed to the inheritance of superior mitochondrial function from mature oocytes to subsequent embryos, which enhances the anti-apoptotic capacity of embryonic cells during rapid cell division. Meanwhile, the expression of pluripotency markers (OCT4, NANOG) and trophectoderm marker (CDX2) in blastocysts was not adversely affected. These findings suggested that cysteine treatment reduced blastocyst apoptosis without impairing normal cell lineage differentiation potential, which is conducive to the acquisition of blastocysts with reduced apoptotic levels for subsequent transplantation.

In conclusion, the core mechanism by which cysteine improves the in vitro maturation quality of bovine oocytes is considered to be a multi-target and synergistic regulatory process. Firstly, exogenous cysteine acts as a synthetic substrate to enhance intracellular GSH synthesis and effectively relieve oxidative stress. Secondly, upregulation of MTHFD1L promotes NADPH production, which serves as a key coenzyme for maintaining the reduced state of GSH and further strengthens the antioxidant system via positive feedback. Thirdly, increased expression of NDUFS2 and VDAC3 enhances the activity of mitochondrial electron transport chain and energy metabolism efficiency. Fourthly, regulation of SCD optimizes lipid metabolism. The coordination of these pathways improves the overall metabolism and internal homeostasis of oocytes, ultimately resulting in higher nuclear maturation rate, superior cytoplasmic quality and enhanced developmental potential.

In summary, these findings suggest that 75 μM as the optimal concentration of cysteine for bovine oocyte in vitro culture, providing clear and reproducible experimental parameters for optimizing bovine in vitro embryo production (IVP) systems. Meanwhile, the underlying mechanism was comprehensively elucidated from the perspectives of redox balance, mitochondrial function and transcriptomic profiling. This work provides novel insights into the regulatory role of antioxidants in female germ cell development.

## 6. Conclusions

Supplementation of 75 μM cysteine in the in vitro maturation medium of bovine oocytes could coordinately improve the redox homeostasis and metabolic status of oocytes through multiple mechanisms, including increasing intracellular GSH level, reducing ROS accumulation, enhancing mitochondrial function, and regulating the expression of genes related to energy and lipid metabolism (NDUFS2, VDAC3, MTHFD1L, SCD, ANXA2). The improvement of cytoplasmic maturation quality ultimately increased the nuclear maturation rate and cleavage rate and produced blastocysts with lower apoptotic level and improved early developmental parameters. The findings provide an important theoretical basis and practical strategy for optimizing the in vitro maturation system of oocytes and improving embryo production efficiency in large domestic animals.

## Figures and Tables

**Table 1:** List of primer information for genetic analysis.

Gene	Primer Sequences (5′-3′)	Size (bp)
*GAPDH*	F:ACGGGAAGCTCACTGGCATGG	227
	R:GCCAGCCCCAGCATCGAAG	
*NDUFS2*	F:GCTGTCATGTACCCCACCAA	167
	R:ATCTCCCCACTCAGCTCCAT	
*VDAC3*	F:GGCTGTGTTGGCCTTTGAAG	125
	R:GAGTGTGCAGCTGGAAGTCT	
*ANXA2*	F:CCAAGTGCATACGGGTCAGT	144
	R:TCTCTGTTCATTGCTGCGGT	
*MTHFD1L*	F:GCTCTGACCGACAGCCTATC	95
	R:AAGTCGTCTGCTGTCACAGG	
*SCD*	F:TTGGAAGAAGACATCCGCCC	165
	R:GCAGGTGGGGATCAATGTGA	

## Data Availability

Data are contained within the article.
